# Associations of parents’ and adolescents’ active travel behavior across various destinations – a sex/gender analysis

**DOI:** 10.1186/s12889-023-15428-x

**Published:** 2023-03-18

**Authors:** Franziska Beck, Isabel Marzi, Denise Renninger, Yolanda Demetriou, Eliane Stephanie Engels, Christina Niermann, Anne Kerstin Reimers

**Affiliations:** 1 Department of Sport Science and Sport, Friedrich-Alexander-Universität Erlangen-Nürnberg, Gebbertstraße 123B, 91058 Erlangen, Germany; 2 Department of Sport and Health Sciences, Technical University of Munich, Georg-Brauchle-Ring 60/62, 80992 Munich, Germany; 3 Institute of Interdisciplinary Exercise Science and Sports Medicine, Medical School Hamburg, Am Kaiserkai 1, 20457 Hamburg, Germany

**Keywords:** Active commuting, Active transport, Youths, Family, Parent-adolescent dyads

## Abstract

**Background:**

Active travel behavior such as walking and cycling is associated with several health benefits. Especially the family environment seems to be important for active travel in children and adolescents. Currently, little is known regarding travel behavior in leisure time and associations of travel behavior within parent-adolescent dyads.

**Methods:**

The present analysis is based on the German ARRIVE study (Active tRavel behavioR in the famIly enVironmEnt), which incorporated a large scale, representative cross-sectional online survey including 517 parent–child dyads consisting of adolescents (*N* = 517; boys = 263, girls = 254) aged 11–15 years and one of their parents (*N* = 517; fathers = 259, mothers = 258). Based on that survey which took place in June 2021 (during the COVID-19 pandemic), we calculated the prevalence of active travel to four commonly visited destinations (school/work, friends/relatives, shopping stores and recreational activities) using an adapted version of the travel to school questionnaire by Segura-Diaz JM, Rojas-Jimenez A, Barranco-Ruiz Y, Murillo-Pardo B, Saucedo-Araujo RG, Aranda-Balboa MJ, et al. (Int J Environ Res Public Health 17(14), 2020). In addition, we investigated the associations between parents’ and adolescents’ travel behavior using scores for school/work, leisure time (friends/relatives, shopping stores and recreational activities) and overall (school/work and leisure time).

**Results:**

Across all destinations, prevalence of active travel in adolescents (63.08%) was higher than in parents (29.21%). Active travel to school (47.33%) as well as to work (20.43%) indicated the lowest prevalence. Linear regression models revealed significant associations in overall active travel between mothers and adolescents (girls: β = 0.308, *p* < 0.001; boys: β = 0.302, *p* = 0.001) and in leisure time active travel behavior between mothers and daughters (β = 0.316, *p* < 0.001). Related to school/work active travel there were no associations between parents and adolescents.

**Conclusion:**

The associations between adolescents’ and parents’ travel behavior differ depending on gender: they are solely seen in mother-adolescents dyads. Furthermore, our findings conclude that travel is a routine and independent of the destination.

## Background

Physical activity plays an important role for adolescents’ health [1]. However, worldwide many adolescents are insufficiently active and do not reach the recommended physical activity of at least 60 min moderate-to-vigorous physical activity [2–4]. Active travel is one option to reduce the lack of physical activity and is defined as using active travel modes (e.g., walking or cycling) for commuting and reaching various nearby destinations in daily life [5, 6]. Active travel is associated with positive health outcomes such as improved cardiovascular fitness, lower cancer-related mortality or reduced weight [7, 8] and mental wellbeing [7, 9]. It also contributes to daily health-enhancing physical activity in adolescents [10]. A twelve-year longitudinal population-based study with adolescents focusing on active travel to school showed positive long-term effects on physical activity behavior in young adulthood during leisure time [11]. In addition to the health benefits stated above, using active travel modes further has a positive impact on the environment due to the low CO2 emissions of these modes [8, 12]. This helps to reduce air pollution which reportedly causes more than half a million deaths every year worldwide [8]. Furthermore, active travel has a wide range of benefits for the social health, in particular through facilitating social interaction with peers [13].

Although active travel is associated with several health and environmental benefits [6, 14], only a small proportion of adolescents walk or cycle to school in Germany and worldwide [15–18]. In Germany, recent representative data from the German MoMo-Study showed that 17.7% of adolescent girls and 20.2% of adolescent boys regularly walk to school and 21.5% of adolescent girls and 25.2% of adolescent boys regularly take their bike [15]. Regarding secular trends in active travel, the percentage of children and adolescents walking or cycling to school declined in most countries [19–21] or remained nearly stable, like in Spain [22]. In summary, the decline in active travel in recent years reinforces the negative effects of physical inactivity and enhances the negative impact on the environment.

Adolescents' health behavior is affected by individual, environmental, and social determinants. Travel behavior is embedded in social contexts, for example peer groups, school, or family environment. Especially the family is important because of its lasting effect on children’s and adolescents’ health related behavior [23, 24]. Since the family environment is the context in which the child begins to develop daily routines, it might determine their future lifestyle choices [25]. Especially parents play an essential role in fostering or hindering physical activity behavior such as active travel in children and adolescents [26–28]. Furthermore, parents are significant reference persons for their children who tend to imitate their social and physical activity behavior [29].

This assumption is mostly operationalized by investigating the association between parent’s and child’s physical activity behavior aiming to provide evidence of resemblance of physical activity in parent–child dyads. For instance Petersen et al. [30] summarized studies with objectively measured child physical activity and found overall a weak positive relationship between parent and child physical activity. This is in line with a meta-analysis by Yao and Rhodes [31] that included studies with objectively and subjectively measured physical activity.

Furthermore, parental behavior may be imitated differently by daughters and sons. Several theories like the cognitive-development theory [32] or the social learning theory [33] suggest that boys and girls are more inclined to imitate behaviors by same-sex models than by opposite-sex models. Studies support the same-sex imitation by demonstrating no difference between boys' and girls' acceptance of the same sex behavior [34, 35]. However, females were more likely than males for opposite-sex choices [34, 35]. Interestingly, the age of the adolescents did not affect these findings [35].

### Associations between parents’ and children’s active travel

With regard to active travel, until now, a few studies have linked parents' active travel with their children's (5–9 years) or adolescents' (10–19 years) active travel. Several studies found a positive association between parents’ and their children’s or adolescents’ active travel [36–39] as well as between the number of steps per day [40, 41]. However, a study from the U.S. found no association between adolescents' and their parents' active travel [42]. The existing studies focused predominantly on the way to school/work. Nevertheless, adolescents as well as their parents travel to more destinations in everyday life [43], and reasons for active travel may vary depending on destination. In particular, in Germany school trips account for 35.5% of trip purposes in the age group of adolescents. Additionally, 39.5% of trips are made related to leisure activities, and 14.5% are related to shopping and everyday accomplishments [44]. Thus, adolescents make a relevant proportion of trips to reach other destinations than schools. In adults aged 30–60 years, a similar contribution can be seen: 37.7% of trips account for work, 14.7% for shopping and 22% for leisure activities [44]. However, until now, only few studies investigated other relevant destinations such as recreational facilities [45, 46]. As a consequence, the investigation of associations between parents’ and adolescents’ travel behavior should be extended to multiple destinations to reflect active travel being a habitual behavior in adolescents and their parents.

Studies addressing sex/gender^1^ differences related to active travel and accounting for dyadic specific differences are rare. In a German study [16], cycling to school in adolescents was related to parents’ cycling behavior to work while further sex/gender analysis showed no significant moderation. Despite this, a study from Portugal revealed that children whose mother, but not father, travels actively to work, were significantly more likely to walk to school [47]. A current study investigating associations of children’s and adolescents’ active travel to school with maternal and paternal active travel found different results in dependence of participants’ age. In children, the study found an association between mothers' active travel with boys’ and girls’ active travel, whereas fathers' active travel was only associated with girls’ active travel [48]. However, active travel in adolescents (mean age = 14.07 years) was not associated with parents’ active travel [48].

Overall, most studies focused on children and studies focusing on adolescents are scarce. There are some differences in the relationship between parents and their children with increase in children’s age though [48]. For instance, shared time of parents and adolescents decreases compared to shared time of parents and children [49]. Therefore, results for children might not be applicable for adolescents.

Thus, the aim of the present study is firstly to assess the prevalence of parents’ and adolescents’ active travel to four commonly visited destinations (school/work, friends/relatives, shopping stores and recreational activities) [50, 51]. Secondly, we want to investigate the association between parents’ and adolescents' active travel behavior separately for leisure time (homes of friends or relatives, shopping, and leisure activities) and to school/work as well as across all destinations. A special focus of the current study is to examine same-sex and opposite-sex associations by investigating associations in travel behavior between different parent-adolescent dyads, namely mother-daughter, father-daughter, mother-son, and father-son.

## Methods

### Study design

The present analysis is part of the ARRIVE study (Active tRavel behavioR in the famIly enVironmEnt), a mixed-method cross-sectional survey in Germany, aiming to gain a deeper understanding of adolescents' active travel behavior by considering a socio-ecological perspective [52]. Data collection took place in times of COVID-19 pandemic from June 17^th^ to June 28^th^ 2021 by means of computer-assisted web interviewing of parents and their adolescents aged 11–15 years. The study was approved by the local Ethics Committee (Ref. No. 249_21B) and was in accordance with the 1964 Declaration of Helsinki. Adolescents provided signed assent and their parents signed informed consent for the study participation.

### Procedure and Participants

The survey made use of an existing nationwide online panel (forsa.omninet) to which access was provided by Forsa, a leading organization for public opinion polls. To be included in this panel, the recruitment took place solely offline via telephone interviewing so that people who use the internet only sparsely are still represented in the sample. The panel is representative for the German population regarding age, sex/gender, education, and place of residence. For the present study, a nationwide balanced sample of mothers and fathers with at least one teenager aged 11–15 years was drawn. In total, 1747 parents were invited to answer the questionnaire of which 518 completed the ARRIVE online survey (response rate: 29.7%). Altogether, data of 518 parents and 518 adolescents was available. One adolescent was excluded prior to analyses because the adolescent indicated a diverse gender and the sample size of *N* = 1 was too small to allow for a separate analysis. The final sample consisted of 517 parent-adolescent dyads, namely mother-daughter (*N* = 127), father-daughter (*N* = 127), mother-son (*N* = 132), and father-son (*N* = 132). After giving informed consent to be contacted for the survey, parents received an invitation e-mail with a link to the questionnaire. Participants were able to answer the survey with a tablet, smartphone, or computer. The online survey was divided into an adult questionnaire and an adolescent questionnaire. After parents completed their part of the questionnaire, they were asked to provide the questionnaire to their teenagers. The survey took the parents about 15–20 min and the adolescents about 10–15 min to complete.

### Measures

The quantitative survey is based on the "Conceptual Framework for the Environmental Determinants of Active Travel in Children" [46]. A detailed description of the framework related to the ARRIVE study can be found elsewhere [52]. The current study focuses on the travel behavior of adolescents and their parents and considers further sociodemographic factors.

#### Active travel

Each of the participants was asked about travel modes typically chosen to reach four different destinations that are considered the most relevant destinations for adolescents which are to school, to friends/relatives, to shopping, and leisure activities [50, 51]. To assess the mode of the destinations, we used a modified version of the mode and frequency of commuting to school questionnaire by Segura-Diaz et al. [53]. This scale was confirmed as a reliable and feasible tool in Spanish adolescents (κ = 0.61–0.94). The questionnaire was translated to German and complemented based on an existing German questionnaire on travel behavior [54]. Thus, participants indicated the usual mode to work (parents) or to and from school (adolescents), to the home of friends or relatives ("Think about the person from your circle of friends or relatives that you visit most often. How do you usually get to this person? Choose the travel mode you mostly travel with"), to shopping and to leisure activities (e.g., parks). Parents and adolescents could answer these questions with "by foot", "by bike", "by e-bike", "by car", "by motorcycle", by bus", "by train /metro", "others" or "I do not travel this way". If participants selected "others", they were asked to specify this answer by typing the used travel mode. A dichotomous variable was built by assigning the travel mode to active (by foot, bike, e-bike) or passive (by car, bus, train/metro, motorcycle) travel mode. Additionally, an overall active travel score was calculated by the proportion of active traveled destinations (ranging from 0 to 4 in parents) and from 0 to 5 in adolescents) in relation to all traveled ways to the assessed destinations (ways traveled actively and passively). Thus, we received values between 0 and 1, indicating the proportion of active traveled destinations. For example, if a mother traveled all four ways (work, friends/relatives, shopping, leisure activities), but only two of them actively, her overall active travel score was 0.5.

$$Overall\ active\ travel\ score= \frac{{N}_{ways\ traveled\ actively}}{{N}_{ways\ traveled\ actively}+{N}_{ways\ traveled\ passively}}$$

To distinguish between leisure travel and school travel, we build a leisure time active travel score by dividing the sum of active travel to friends/relatives, to shopping, and leisure time by the sum of all destinations the participant traveled to/provided answers for. In that way, we were able to account for destinations participants did not travel to at all.

$$Leisure\ time \left(to\ friends/relatives,\ shopping,\ leisure\ activities\right) active\ travel\ score= \frac{{N}_{ways\ traveled\ actively\ to\ the\ three\ destinations}}{{N}_{ways\ traveled\ actively\ to\ the\ three\ destinations}+{N}_{ways\ traveled\ passively\ to\ the\ three\ destinations}}$$

Similarly, a mean school travel score was built including the transport mode to and from school.

$$School \left(to\ and\ from\right) active\ travel\ score= \frac{{N}_{ways\ traveled\ actively\ to\ and\ from\ school}}{{N}_{ways\ traveled\ actively\ to\ and\ from\ school}+{N}_{ways\ traveled\ passively\ to\ and\ from\ school}}$$

In addition, travel distance was assessed. Distance to school was obtained from parental questionnaire, and distance to the other destinations was investigated via adolescents’ self-reports [53]. This scale is part of the questionnaire of Segura-Diaz et al. [53] and shows very good reliability (κ = 0.9). Adolescents could select between less than 500 m, between 500 m and 1 km, between 1 and 2 km, between 2 and 3 km, between 3 and 5 km, and more than 5 km. To be included in the analysis, we build a mean value of each range of distance.

#### Sociodemographics

Parents reported sociodemographic data such as age and sex/gender of themselves and of their child. Furthermore, parents indicated the body weight and height as well as the school type of their child. The type of urbanization was determined according to the population of the participants' hometowns. Having more than 100,000 inhabitants was categorized as cities, medium-sized towns included 20,000–99,999 inhabitants, hometowns with 5,000–19,999 inhabitants were coded as small towns and rural areas consisted of less than 5,000 inhabitants [55].

### Data analysis

All statistical tests were conducted using IBM SPSS 26 (IBM Corporation, Armonk, NY, USA). For all variables, < 5% of values were missing. Missing data were listwise deleted after checking that missing values were completely at random using Little’s MCAR test (*p* = 0.154) [56]. Descriptive statistics were calculated for study variables, mean (M) and standard deviations (SD) for continuous variables and frequency (%) for categorical variables. The comparison of categorical variables (prevalence of travel mode across all destinations) according to sex/gender in parents (mothers and fathers) and adolescents (boys and girls) was examined using Pearson-Chi2-Test.

To examine associations between adolescents and parental active travel behavior, separate sex/gender analyses with parent-adolescent dyads (mother-daughter, mother-son, father-daughter, father-son) were conducted by linear regression models. For each travel score, namely school/work, leisure time and overall, the dependent variable was the proportion of active travel of adolescents (boys or girls) and the independent variable was the parental active travel proportion (mother or father). All regression models were controlled for adolescents' weight status, school type, distance to destination, and type of urbanization. To account for multiple testing, Bonferroni corrected α -level (α = 0.004) was set as a threshold to determine statistical significance.

## Results

### Characteristics of the study population

In total, 517 parent-adolescent dyads completed the survey (258 mothers and 259 fathers; 254 girls and 263 boys). Sociodemographic characteristics of the participants are presented in Table 1. Mean age was 47.67 years (SD = 5.28) for parents and 13.07 years (SD = 1.35) for adolescents.

**Table 1:** Sociodemographic characteristics of parents and adolescents

**Parents**	**Overall**	**Mothers**	**Fathers**
N (%)	517 (100)	258 (49.9)	259 (50.1)
Age (M; SD)	47.67 (5.28)	46.21 (4.83)	49.14 (5.30)
**Residential Area (N;%)**			
City > 100,000 inhabitants	154 (29.2%)	76 (29.5%)	75 (29.9%)
Medium sized town 20,000–100,000 inhabitants	90 (17.4%)	44 (17.1%)	46 (17.8%)
Small town 5,000–20,000 inhabitants	115 (22.2%)	56 (21.7%)	59 (22.8%)
Rural area/village	159 (30.8%)	80 (31.0%)	79 (30.5%)
**Adolescents**	**Overall**	**Girls**	**Boys**
N (%)	517 (100)	254 (49.1)	263 (50.9)
Age (M; SD)	13.07 (1.35)	12.92 (1.35)	13.21 (1.33)
Weight status (BMI) (M; SD)	19.23 (3.32)	19.12 (3.09)	19.33 (3.56)
**School type (N;%)**			
Elementary school	12 (2.3%)	8 (3.1%)	4 (1.5%)
Middle school	24 (4.6%)	11 (4.3%)	13 (4.9%)
Secondary school	99 (19.1%)	44 (17.3%)	55 (20.9%)
High school	294 (56.9%)	154 (60.6%)	140 (53.2%)
Comprehensive school	74 (14.3%)	31 (12.2%)	43 (16.3%)
Internat	2 (0.4%)	2 (0.8%)	0
Others	8 (1.5%)	3 (1.2%)	5 (1.9%)

### Distribution of travel mode choice

The percentage of active travelers across the destinations separately for sex/gender groups is presented in Table 2. There was a significant difference between boys and girls in the travel modes to friends (*p* = 0.011) and for shopping (*p* = 0.002). In parents, there were no significant differences in travel modes between mothers and fathers. Regarding adolescents’ travel mode, boys and girls most often traveled actively to friends (72.9%) and for shopping (73.6%). The proportion of adolescents traveling actively vs. non-actively to and from school is comparable. Across all destinations, the percentage of passive travelers among parents was higher than the percentage of active travelers.

**Table 2:** Distance and percentage of active travelers across the four destinations in adolescents and parents. Distance [km] = mean (SD)

	Girls	Boys	*p*-value	Mother	Father	*p*-value
To school/work						
Distance [km]	6.4 (5.7)	6.4 (5.8)		16.9 (18.1)	22.2 (14.8)	
Active travel [%]	43.70	50.95	0.099	21.10	19.76	0.731
From school						
Distance [km]	6.4 (5.7)	6.4 (5.8)				
Active travel [%]	44.49	50.57	0.166			
Friends						
Distance [km]	2.4 (1.7)	2.5 (1.6)		11.7 (26.7)	17.9 (36.6)	0.103
Active travel [%]	67.86	77.78	*0.011*	46.09	38.85	
Shopping						
Distance [km]	2.0 (1.6)	2.0 (1.4)		4.3 (4.4)	4.5 (4.0)	0.393
Active travel [%]	67.35	79.53	*0.002*	23.26	20.08	
Leisure activities						
Distance [km]	2.5 (1.6)	2.5 (1.5)		8.1 (13.8)	11.6 (21.3)	0.528
Active travel [%]	65.31	73.23	0.055	39.76	42.35	
Overall						
Active travel [%]	57.74	66.41		30.26	28.16	

### Associations in travel behavior between parents and adolescents

Results of the linear regression models analysing the associations between parents and adolescents regarding active travel overall, active travel to work/school and active travel in leisure time can be seen in Table 3.

**Table 3:** Associations in active travel behavior between parents and adolescents

		Mother			Father	
	**β**	**T**	***p*-value**	**β**	**T**	***p*-value**
School/work						
Girls	0.103	1.088	0.279	0.169	2.058	0.042
Boys	0.303	2.776	0.007	0.029	0.255	0.800
Leisure time						
Girls	0.316^a^	4.243	< 0.001	0.232	2.592	0.011
Boys	0.188	2.451	0.016	0.004	0.053	0.958
overall						
Girls	0.308^a^	4.39	< 0.001	0.237	2.429	0.017
Boys	0.302^a^	3.366	0.001	0.037	0.39	0.698

^a^ Bonferroni corrected α -level (α = 0.004); Linear regression models controlled for BMI of the adolescents, adolescents' distance to destinations, school type, and type of urbanization

No association was found between active travel behavior to/from school respectively work in adolescents and their parents. For leisure time travel, only the association between mothers’ and girls’ active travel behavior reached significance (β = 0.316, T-value = 4.243). Adolescents’ overall active travel behavior is associated with the overall active travel behavior of their mothers (girls: β = 0.308, T-value = 4.39; boys: β = 0.302, T-value = 3.366). Fathers active travel behavior was not related to adolescents behavior, neither for boys nor for girls and for none of the three travel scores.

## Discussion

In this study we examined the prevalence of active travel to four commonly visited destinations (school/work, friends/relatives, shopping stores and recreational activities) in parents and adolescents. Furthermore, we investigated the dyadic associations between parents’(mothers’ and fathers’) and adolescents’ (girls’ and boys’) active travel behavior to work/school, in leisure time and overall in a representative sample from Germany accounting for different sex/gender specific parent-adolescent dyads (mother-daughter, mother-son, father-daughter, father-son). In our sample, about 60% of adolescents and only 30% of parents regularly used active travel modes to reach the four destinations (school/work, friends/relatives, shopping and recreational activities). Across all travel scores, fathers' active travel behavior was not associated with adolescents' active travel behavior whereas significant associations were found between mothers’ and adolescents’ overall active travel behavior and between mothers’ and girls’ leisure time travel behavior. For school/work-related traveling, no associations between parents’ and adolescents’ behavior were found.

### School/work travel behavior

Travel to school/work represents a daily routine. However, twice as many adolescents traveled actively to school than parents traveled actively to work. This tendency is in line with the studies of Aibar Solana et al. [37] and Schönbach et al. [16] whereas another study from Spain and Chile revealed that almost as many adolescents as parents choose an active travel mode to school and work, respectively [48]. This differences could be explained by the urban vs. rural environments where the study population living mostly in cities (Granada and Valparaiso) which is related with higher percentages of active travelers compared to rural areas [15, 57, 58]. In the present study, participants of urban and rural environments were included. However, we did not differentiate according to urbanization level. However, we found that travel distance to work is larger than the travel distance to school. This might explain the difference in prevalence of active travel behavior between parents and adolescents.

In the present study, associations in active travel behavior to school/work between parents and adolescents were not significant, neither for mothers nor for fathers. Similarly, data from the U.S. found no associations between parents’ general travel behavior and adolescents’ travel to and from school [42]. Additionally, a recent study that investigated the associations between adolescents’ travel behavior to school and their mothers’ and fathers’ travel to work found no associations [48]. In contrast, significant associations were found for children’s and parents’ school/work related travel behavior [48]. This difference could be explained by a higher degree of autonomy and independence of adolescents [59–61]. Additionally, parents perceive fewer barriers to active travel with regard to with increasing age of their child [62, 63]. As a consequence, adolescents seem to predominantly choose their preferred travel mode independently from their parents [64]. However, Brand et al. [25] found associations between adolescents’ active travel to school and mothers’ active travel to work and Rodriguez-Rodriguez et al. [65] found that fathers’ active commuting to work in adolescents were important variables for explaining active commuting to school.

### Leisure time travel behavior

Contrarily to the way to and from school, traveling to friends, shopping opportunities, or leisure activities does not occur on a regular and daily basis in most adolescents [43]. Thus, travel mode choice might differ between these leisure activities and transport mode choice to work and school. In our study, more adolescents choose an active travel mode for non-school destinations than for their way to and from school. For parents, the prevalence of active travel doubled for leisure activities and visiting friends in comparison to work. The travel modes for shopping indicated the highest differences between parents and adolescents while 74% of adolescents chose an active travel mode for shopping only 22% of parents did so. However, shopping opportunities for both, parents and adolescents, were within a feasible walking respectively cycling distance (2 km and 4 km) [66, 67]. The differences in active travel to shopping facilities between parents and adolescents could be explained by the fact, that shopping in adolescence serves as an opportunity to be with friends and thus, adolescents might predominantly socialize or simply take a respite from adult supervision [68]. In contrast, parents’ shopping is often related to shopping for groceries [43] which may result in heavy stuff to carry and thus, may imply using the car or bus to go shopping.

Regarding leisure time active travel behavior, our analysis indicated only a significant association between mothers and their daughters. This result might be explained by a different socialization between boys and girls. First of all, literature suggests that boys start at an earlier age to be more autonomous and independent of their parents than girls [61]. Thus, they try to distance themselves from their parents by not meeting parental expectations, which might also apply for travel behavior. Furthermore, studies indicate a greater emotional connection between mothers and daughters in adolescence [69]. Additionally, adolescent sons are granted more independence and freedom [70] while higher safety concerns seem to deter girls from active travel, specifically from cycling [71]. This is in line with our findings of a slightly lower prevalence in leisure time active travel in girls and significant gender differences between boys and girls in active travel to friends and to shopping opportunities.

### Overall travel behavior

Contrary to the travel behavior in leisure time that indicated only significant associations between mothers and daughters, overall active travel behavior including all destinations revealed associations between mothers and both, boys and girls. This indicates that the parent-adolescent associations of travel behavior are less destination specific but rather reflect a general tendency to choose an active travel mode within the family. Underlying mechanisms might collective attitudes or behavioral habits that are incorporated into daily routines [72]. Therefore it can be concluded that the likelihood that adolescents develop active travel routines is higher if their parents, especially mothers, are active travelers. An existing study confirmed this assumption by investigating the association between adolescents’ travel to school and any vs. no active travel in parents in everyday life. Results indicated positive associations between parents’ and adolescents’ travel behavior [38]. These findings emphasize the assumption that the active travel behavior is a generalized behavioral tendency that seems to be highly stable across different destinations.

### Sex/gender analysis

With regard to the sex/gender focus in the present study, our results indicate that mothers' travel behavior is more relevant for adolescents’ transport mode choice than paternal behavior as only mothers’ travel behavior is associated with adolescents’ active travel behavior. This could be explained by the fact, that still mothers seem to be the primary caregivers spending more time supervising the children compared to fathers [73, 74]. Even if fathers also spend time together with their children, the amount is lower compared to mothers [75]. In Germany, mothers predominantly work part-time and are responsible for the household and child care [76], resulting in spending more time with the children. Especially during the Covid-19 pandemic, these gender roles within a family were strengthened [77] and thus, mothers are more visible to adolescents than fathers. Additionally, the predominance of mother-adolescent associations could be explained by the fact that fathers reported longer travel distances to all destinations than mothers, which is associated with a higher rate of passive travel modes among fathers.

The visibility of active travel behavior seems to be a critical issue. According to Larsen et al. [78], parental behavior needs to occur directly in front of the child to enable an imitation effect in terms of modeling. However, studies examining the association between parent and child physical activity mostly do not distinguish between parent behavior that takes place in front or in absence of the children. It is mostly unknown to what extent parent’s physical activity takes place in front of the child. This applies for our study as well, we have no information to what extent parents’ travel behavior was visible to their children. However, it is important to distinguish between parental physical activity and parental physical activity modeling. Furthermore, two types of physical activity modeling could be distinguished: on the one hand ‘role modeling’ that is a parenting practice and displays an active and intentional process. The behavior is intentionally demonstrated in front of the child aiming to affect the child’s behavior. On the other hand, ‘modeling’ of a behavior takes place in front of the child and might be imitated as well but the behavior is not intentionally performed [78, 79]. While the former is typically related to “healthy” behaviors such as being physically active, the latter relates to both healthy and unhealthy behaviors such as sedentary behavior. However, although this distinction conceptually and theoretically makes sense, empirically differentiating between these types of modeling is challenging and should be addressed in future studies. Furthermore, it should be noted that this distinction might be useful to explain inconsistencies in study findings and overall small effect sizes regarding the associations between parents’ and their children’s behavior.

Having all findings in mind, parents and especially mothers should be encouraged use active travel modes as often as possible, particularly when travel is visible to their children. In light of low active travel prevalence in parents, strategies are needed that motivate parents and support them in developing active travel routines. As proposed by Sallis et al. [80] and stated within the Global Action Plan on Physical Activity 2018–2030 [81] not only individual factors need to be considered in the promotion of physical activity. Further, the policy is required to act and provide an activity, friendly environment that encourages active travel behavior for all, children and adults. This firstly reduces passive travel and thus, is associated with several health benefits [7, 8] and addresses current global health challenges such as climate change by switching to sustainable transport modes. However, as the behavioral associations between parents and their children are not unidirectional but reciprocal within families (see for example family-as-system approaches [82]), the promotion of adolescents’ active travel might also be a good starting point to affect adults active travel behavior.

In summary, our study widens the current view on active travel behavior in adolescents and parents by focusing not only the way to and from school/work, but also including leisure time related destinations. Furthermore, we analyzed sex/gender specific dyadic associations between parent and adolescent travel mode choice. Further research should also take into account the question to what extent parents’ travel behavior is visible for the children and how relevant this visibility is. Additionally, the difference between intentional and non-intentional behavior should be addressed. Furthermore, active travel includes various kinds of travel modes, namely, walking, cycling, and other non-motorized modes of transport, and thus, further research should analyze associations separately for different transport modes and compare them. For example, to non-school destinations, boys are more likely to cycle than girls [83], and men use the bicycle more frequently to travel to leisure activities than women [43]. To the best of our knowledge, Schönbach et al. [16] is the only study analyzing the association between cycling to school and work in adolescents and their parents and found declining odds for cycling to school in adolescents whose parents did not cycle to work. This could help to deepen the understanding of associations and could help to promote active travel behavior within the family. To better understand the influence of the family on children’s and adolescents’ active travel behavior, further studies should include both, mothers and fathers, and other potential persons, such as siblings or grandparents.

### Strength & Limitations

The present study has some notable strengths. First, the sample is drawn from a nationwide representative sample with regard to age, sex/gender, level of education and living area and focused on an age group with a high-risk of physical inactivity [4]. This sampling procedure enabled a balanced selection of mothers and fathers having at least one child aged 11–15 years allowing the investigation of mother–child and father-child dyads. Finally, in contrast to existing active travel research, the present study focused not only on active travel to school, but also on destinations traveled to in leisure time.

Nevertheless, there are some limitations that have to be mentioned. First, the design of the study was cross-sectional and therefore, no conclusions on direction and causality can be drawn. Subjective measures and self-reported data indicate a limitation, since it might not be free of recall bias and social desirability. Furthermore, data were obtained only from one parent, thus, no conclusion about both parents can be made. In addition, our study could not differentiate between different types of modelling (intentionally vs. unintentionally). A further limitation refers to the associations between parents and adolescents. We solely investigated association within the same destinations and did not focus on associations in travel behavior across different destinations in parents and adolescents (e.g. school travel in adolescents and leisure time travel in parents). Finally, it should be mentioned, that data collection took place during the Covid-19 pandemic. Even though in summer 2021 there were no restrictions regarding the assessed destinations, we cannot preclude an impact of the pandemic on travel behavior in adolescents and their parents.

## Conclusion

The present study provides nationwide data on active travel behavior in adolescents aged 11–15 years and their parents from Germany. In addition, associations in active travel behavior across different destinations between adolescents and their mothers/fathers were investigated. Overall, a high percentage of adolescents in our study traveled actively whereas mothers and fathers mostly traveled passively. Furthermore, active travel associations are more likely to exist between mothers and adolescents than between fathers and adolescents. Dyadic associations seem not to be destination-specific rather than to have an overall tendency to choose actives vs. non-active travel modes within the dyads. As it is unclear whether these associations are a result of (role) modeling, further research should focus on distinguishing between parents’ behavior, unintentional modeling and intentional role modeling and include the aspect of visibility of parents’ active travel behavior and its intentionality. Practical implications refer to the promotion of active travel of parents and their children by providing an activity-friendly environment for all family members.

## Data Availability

The datasets used and/or analyzed during the current study are not publicly available because informed consent from study participants or their legal guardian(s) did not cover public deposition of data but are available from the corresponding author on reasonable request.

## Footnotes

1 The Cochrane Sex/Gender Methods Group, a subgroup of the Campbell and Cochrane Equity Methods Group, emphasizes that sex-based biological factors and gendered social factors influence each other and inter-actively shape health behaviour, opportunities and outcomes. In recognition of this theoretical and empirical entanglement, the group recommends using the term sex/gender [84]. Sex/Gender Methods Group. Why sex and gender matter in health research synthesis [Available from: http://methods.cochrane.org/equity/sex-andgender-analysis, [85]. Springer KW, Mager Stellman J, Jordan-Young RM. Beyond a catalogue of differences: a theoretical frame and good practice guidelines for researching sex/gender in human health. Social science & medicine (1982). 2012;74(11):1817–24. We adopted this in the present study.
